# Ligament reconstruction using a semitendinosus tendon graft for proximal tibiofibular joint disorder: Case report

**DOI:** 10.1051/sicotj/2022008

**Published:** 2022-04-01

**Authors:** Atsushi Okubo, Yoshiteru Kajikawa, Shun Nakajima, Nobuyoshi Watanabe, Tadahiko Yotsumoto, Yasushi Oshima, Norishige Iizawa, Tokifumi Majima

**Affiliations:** 1 Department of Orthopaedic Surgery, Kyoto Kujo Hospital 10 Karahashi Rajomon, Minami-ku Kyoto Kyoto 601-8453 Japan; 2 Department of Orthopaedic Surgery, Nippon Medical School 1-1-5 Sendagi Bunkyo-ku Tokyo 113-8602 Japan

**Keywords:** Tibiofibular joint, Semitendinosus tendon, Ligament, Reconstruction

## Abstract

We report our case of ligament reconstruction for treatment of proximal tibiofibular joint disorder using a semitendinosus tendon graft. A 21-year-old male college soccer player with no remarkable history of injury had been suffering from pain at the lateral aspect of the left knee when playing soccer. At another hospital, the patient was diagnosed with a lateral meniscus injury and cartilage injury of the external condyle of the tibia and underwent partial resection of the meniscus and bone drilling. However, his symptoms continued, and he was referred to our institution. Instability of the left proximal tibiofibular joint and pain were noted during weight-bearing dorsiflexion of the ankle. We diagnosed the case as a proximal tibiofibular joint disorder and surgically treated it by dissecting the proximal portion of the semitendinosus tendon, creating one transfibular and two transtibial tunnels, and then reconstructing the proximal tibiofibular ligament using the harvested semitendinosus tendon graft. The patient was allowed to run at postoperative 2 months, with no pain occurring while squatting at postoperative 3 months, subsequently resuming soccer at postoperative 8 months. The proximal tibiofibular joint disorder is a relatively rare pathology, and diagnosis and conservative treatment are often difficult. Although various surgical treatments are known, the clinical outcome of our case has been successful after reconstructing the anterior and posterior proximal tibiofibular ligaments using a semitendinosus tendon graft.

## Introduction

The proximal tibiofibular joint disorder is a relatively rare pathology, and diagnosis and appropriate treatment methods are yet to be established. Herein we report a case of successfully treated proximal tibiofibular joint disorder of a college soccer player who had no obvious history of injury by means of ligament reconstruction using the semitendinosus tendon.

## Case report

A 21-year-old male felt pain in the lateral aspect of the left knee while playing soccer which lasted for 3 months, causing him to consult an orthopaedic clinic. He was diagnosed with lateral medial meniscus injury and cartilage injury of the lateral condyle of the tibia, and he underwent partial resection of the meniscus and bone drilling; however, his symptoms continued, and he was referred to our institution.

**Figure 1:**
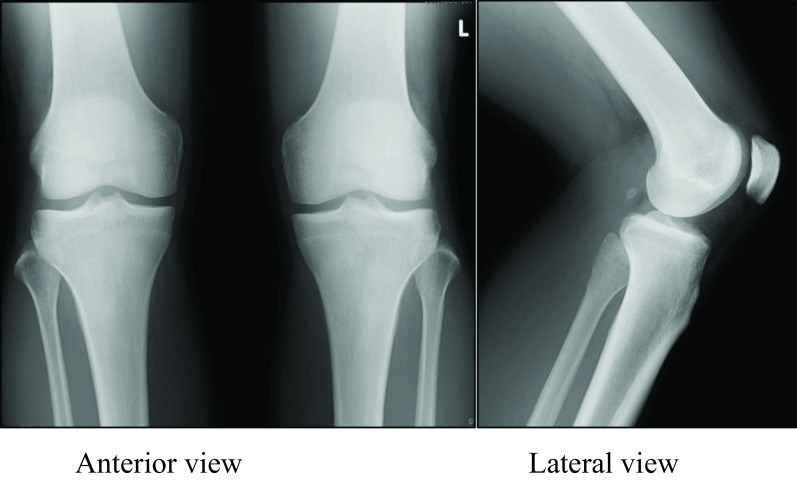
Plain X-ray images at the first consultation. Inclination angle of proximal tibiofibular joint was 26° (based on the measurement method of Ogden), showing an oblique type, with no notable articular degeneration of the proximal tibiofibular joint.

**Figure 2:**
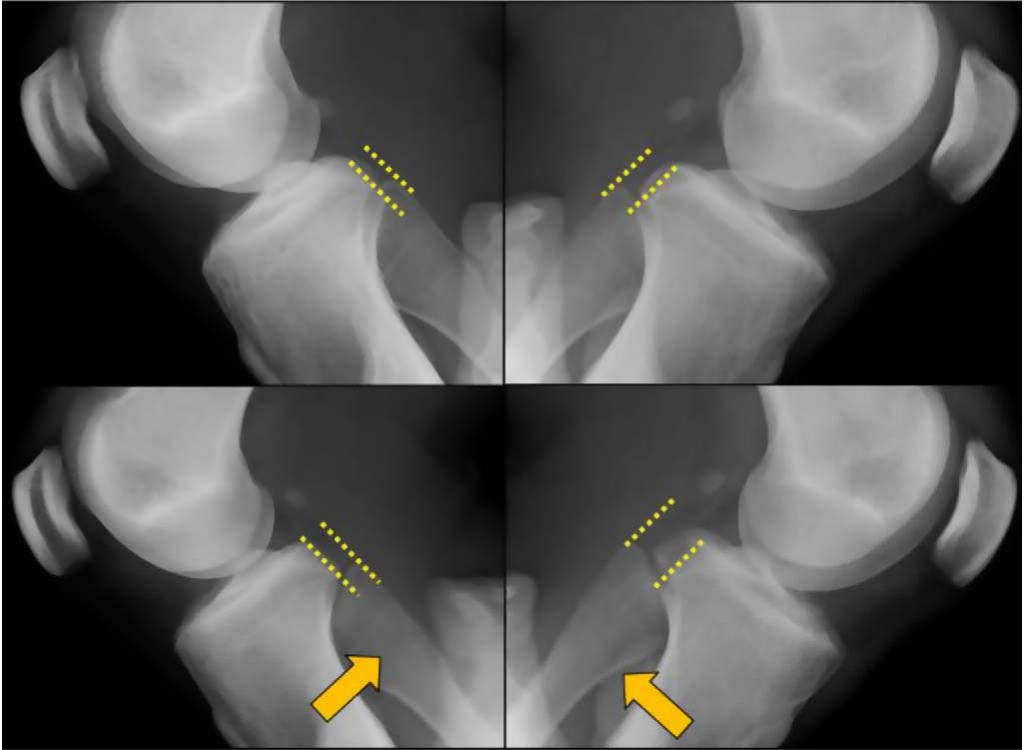
Anteroposterior stress X-ray imaging of the tibiofibular joint. Difference between the right and left sides are seen on anteroposterior stress X-ray images of the tibiofibular joint.

**Figure 3:**
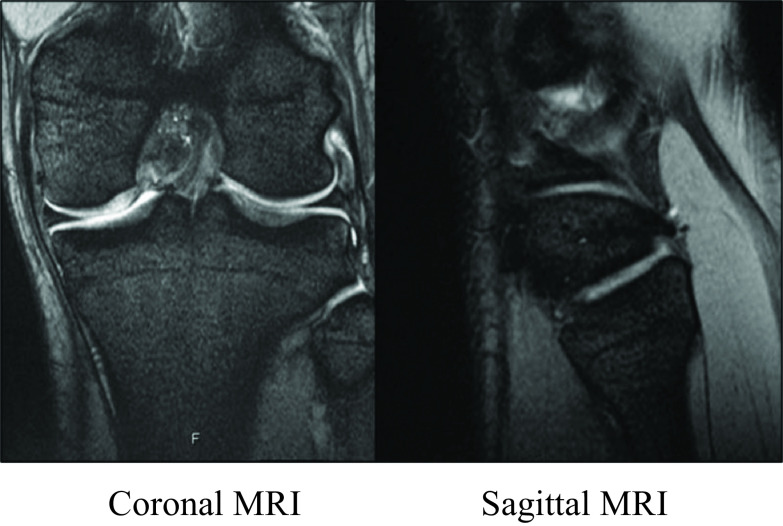
T2-weighted MRI findings. No obvious lateral meniscus injury and cartilage injury was shown on T2-weighted MRI.

Upon the first consultation at our orthopaedic department, the pain was noted when bending the knee (including squatting); additionally, pain occurred in the proximal tibiofibular joint during dorsiflexion of the ankle, and general joint laxity (GJL) was not observed. ROM of the left knee joint was 0° at extension and 140° at flexion. Anteroposterior instability was noted when touching manually at the superior tibiofibular joint. No obvious neurological or cardiovascular disorder was noted. The pre-operative Lysholm score was 68, and the Tegner score was 5. Pre-operative plain X-ray findings showed the inclination angle of Ogden was 26° at the proximal portion of the superior tibiofibular joint, suggesting an oblique type. No obvious arthropathic change was observed at the proximal tibiofibular joint (Figure 1). A difference between the right and left superior tibiofibular joints was observed on anteroposterior stress X-ray images (Figure 2). T2-weighted MRI did not show notable lateral medial meniscus injury or cartilage injury (Figure 3). We diagnosed this case as a left proximal tibiofibular joint disorder based on anteroposterior instability with a right–left difference of the tibiofibular joint and reproducible pain at the proximal tibiofibular joint during weight-bearing dorsiflexion of the ankle.

Initially, conservative exercise therapy and steroid injections were done for 3 months, but the lesion did not show improvement. Thus, we decided to perform ligament reconstruction using a semitendinosus tendon graft. Only the proximal portion of the semitendinosus tendon was dissected, and the pes anserinus was preserved.

**Figure 4:**
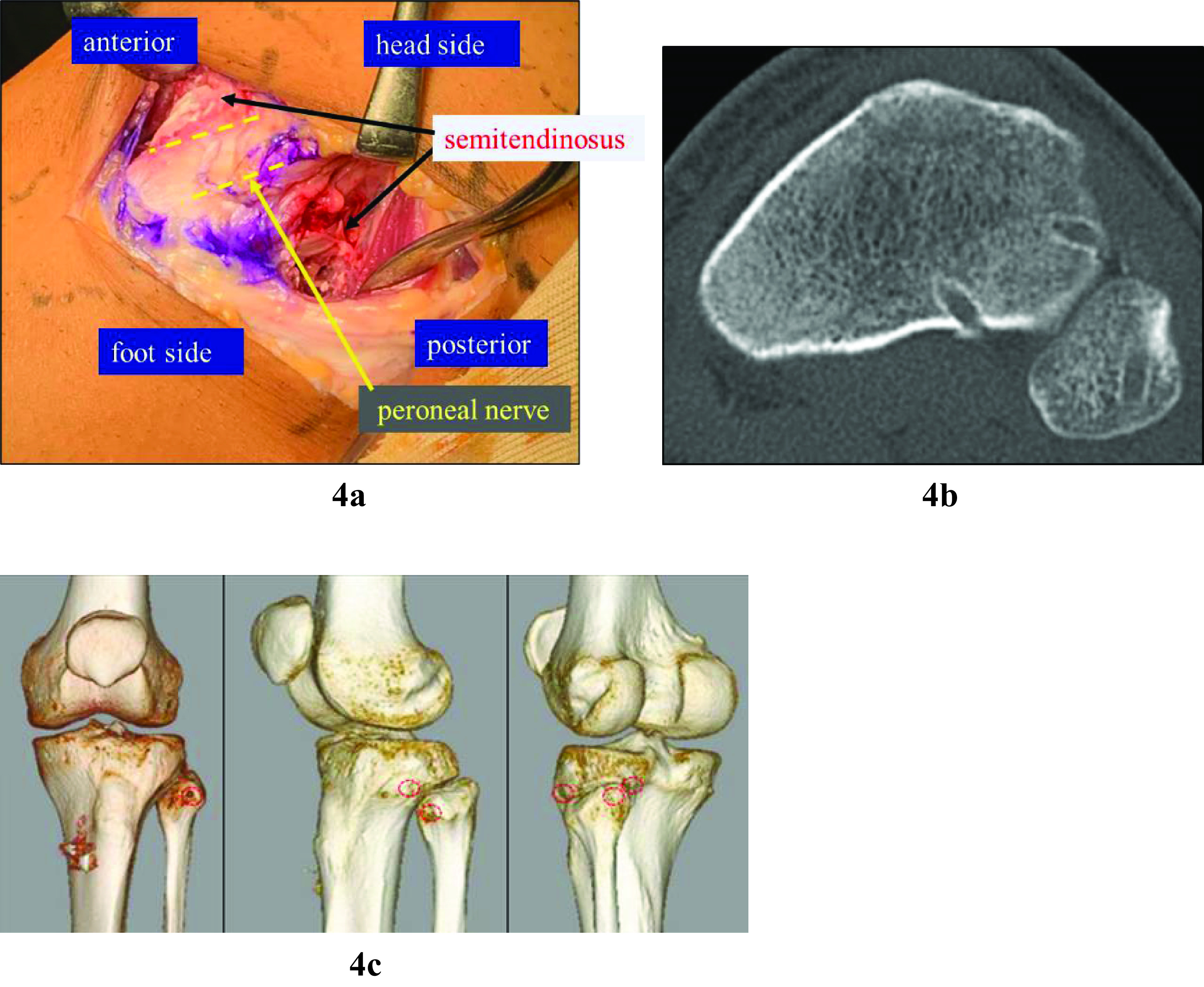
Operative method. A semitendinosus tendon graft was collected. Oblique skin incision was made centring the fibular head and opened, confirming the common fibular nerve. Bone tunnels were made according to the method described by Kobbe et al. [1]. The obtained semitendinosus tendon was passed through the transtibial and transfibular tunnels and fixed to the pes anserinus using a stapler with maximum manual force at the knee joint flexion angle of 30°.

An oblique skin incision was made centring the fibular head, then opened to confirm the location of the common peroneal nerve. Two transtibial and one transfibular tunnel, each 4.5 mm in diameter, were made according to the methods reported by Kobbe et al. [1]. The semitendinosus tendon graft was passed through the tunnels and fixed to the pes anserinus using a stapler with maximum manual force at the knee flexion angle of 30°; then, the anterior and posterior proximal tibiofibular ligaments were reconstructed (Figure 4).

Postoperatively, an extension orthosis was applied for two weeks, avoiding bearing weight on the left lower limb. Partial weight-bearing was started at postoperative 3 weeks and full weight-bearing at postoperative 5 weeks. At postoperative 6 weeks, ROM exercise was started without restrictions. The patient started jogging at 2 months and resumed playing soccer at 8 months postoperatively. A 13-month postoperative examination confirmed an improved condition, showing ROM of 0° at extension, 150° at flexion, Lysholm score of 85, and Tegner score of 9.

## Discussion

Accurate diagnosis of proximal tibiofibular joint disorder was difficult for our case as there was no history of injury, and there is no established consensus on the diagnosis. Our diagnosis of proximal tibiofibular joint disorder was based on the presence of pain at the lateral aspect of the left knee during flexion of the knee joint and dorsiflexion of the ankle while bearing weight, resultant pain around the proximal tibiofibular ligaments, and, finally, on the anteroposterior stress X-ray images of the proximal tibiofibular joint showing a right/left difference. As conservative treatment was ineffective for our case, we performed a reconstruction of the anterior and posterior proximal tibiofibular ligaments using a semitendinosus tendon graft according to the methods of Kobbe et al. [1], and the patient was able to play soccer at postoperative 8 months.

Causes of pain in the lateral aspect of the knee include lateral meniscus injury, iliotibial band inflammation, lateral collateral ligament injury, and proximal tibiofibular joint disorder, the latter of which is relatively rare. In the proximal tibiofibular joint, the fibula moves outward by abduction at the time of dorsiflexion of the ankle, and the rotation forces at the ankle are dispersed. At the knee joint flexion position, the biceps femoris muscle and lateral collateral ligament are relaxed, and the head of the fibula moves forward. In contrast, at the knee joint extension position, tension occurs at the biceps femoris muscle and lateral collateral ligament thus, the head of the fibula moves backwards. If there is instability of the proximal tibiofibular joint, the fibula is believed to move a greater distance than normal, relative to the dorsiflexion of the ankle and bending/stretching movements of the knee joint. The proximal tibiofibular joint consists of the anterior proximal tibiofibular ligament (APTFL) and posterior proximal tibiofibular ligament (PPTFL). The APTFL is comprised of 3 bundles, being thick with great strength, whereas the PPTFL is weaker as it is a wide bundle [2]. It has been reported that APTFL controls the dorsiflexion and abduction of the ankle while PPTFL controls plantar flexion or adduction of the ankle and knee flexion [3]. Ogden JA classified the proximal tibiofibular joint as a horizontal type when the inclination angle is 20° or less, and as an oblique type when the inclination angle is more than 20°, and that the incidence of the proximal tibiofibular joint disorder is high in the oblique type, as was observed in our case. Diagnosis of proximal tibiofibular joint disorder with no notable injury history is often difficult; thus, the possibility of other pathologies which involve pain in the lateral aspect of the knee should first be excluded. Careful consultation is considered necessary for diagnosing pressure pain localised in the proximal tibiofibular ligament or pain in the lateral side of the knee, which occur at dorsiflexion of the ankle and when bending the knee joint. Treatment methods of the proximal tibiofibular joint disorder include conservative treatment and the use of a tibiofibular band. Also, various surgical procedures have been reported, including fibular head resection, a combination of arthroplasty and osteotomy of the fibula, and repair or reconstruction of the tibiofibular ligament. Previous studies have reported that clinical symptoms continued in 23.1% of patients treated by conservative treatment. The incidence of complications was lower after ligament reconstruction than after arthroplasty or fibular head resection [4]. Fibular head resection may induce posterior-lateral instability, thus is not recommended for athletes. The femoral biceps tendon, an iliotibial band fascial graft, and the semitendinosus tendon are recognised as materials for ligament reconstruction. However, there are risks of invasiveness to the soft tissue and damaging the lateral knee stability system if ligament reconstruction was done by splitting the femoral biceps tendon or the iliotibial band [1, 5].

Our case was correctly diagnosed as a proximal tibiofibular joint disorder. In choosing a surgical method, we excluded fibular head resection, as it is unsuitable for an athlete due to the possibility of postoperative posterolateral instability. To avoid that complication, we selected ligament reconstruction of the anterior and posterior ligaments for treatment of the proximal tibiofibular joint ligament disorder, as described by Kobbe et al., with a resultant favourable outcome [1].

## Conclusion

The proximal tibiofibular joint disorder is a relatively rare pathology, for which accurate diagnosis and effective conservative treatment are sometimes difficult. While various surgical methods have been reported for its treatment, we selected anterior and posterior tibiofibular ligament reconstruction using a semitendinosus tendon graft, and a favourable treatment outcome was achieved.

## Conflict of interest

The authors declare that they have no conflicts of interest regarding the present study.

## Funding

The authors received no special funding related to this study.

## Ethical approval

The institutional review board of Kujo Hospital, Japan, approved to conduct this study.

## Informed consent

The subject was explained and signed a written consent form.

## Authors’ contributions

Atsushi Okubo acquired data and analysed results. Yoshiteru Kajikawa provided clinical advice and critically revised the contents of the manuscript. Nobuyoshi Watanabe, Tadahiko Yotsumoto, and Shun Nakajima participated in the clinical treatment of patients and the improvement of this manuscript. Yasushi Oshima and Norishige Iizawa made critical comments on the contents of the manuscript and assisted in the completion of this manuscript. Tokifumi Majima comprehensively reviewed and gave suggestions to improve this manuscript.

## Consent for publication

The subject was explained and signed a written consent form.

## Availability of data and materials

The datasets used and/or analysed during the current study are available from the corresponding author on reasonable request.

## References

[1] Kobbe P, Flohe S, Wellmann M, et al. (2010) Stabilization of chronic proximal tibiofibular joint instability with a semitendinosus graft. Acta Orthop Belg 76, 830–833. 21302584

[2] Anavian J, Marchetti DC, Moatshe G, et al. (2018) The forgotten joint: Quantifying the anatomy of the proximal tibiofibular joint. Knee Surg Sport Traumatol Arthrosc 26, 1096–1103. 10.1007/s00167-017-4508-8 28321475

[3] Alves-da-Silva T, Guerra-Pinto F, Matias R, et al. (2019) Kinematics of the proximal tibiofibular joint is influenced by ligament integrity, knee and ankle mobility: An exploratory cadaver study. Knee Surg Sport Traumatol Arthrosc 27, 405–411. 10.1007/s00167-018-5070-8 30056605

[4] Kruckeberg BM, Cinque ME, Moatshe G, et al. (2017) Proximal tibiofibular joint instability and treatment approaches: A systematic review of the literature. Arthrosc – J Arthrosc Relat Surg 33, 1743–1751. 10.1016/j.arthro.2017.03.027 28865578

[5] Sekiya JK, Kuhn JE (2003) Instability of the proximal tibiofibular joint. J Am Acad Orthop Surg 11, 120–128. 12670138 10.5435/00124635-200303000-00006

